# Pharmacokinetics and dose adjustment of etoposide administered in a medium-dose etoposide, cyclophosphamide and total body irradiation regimen before allogeneic hematopoietic stem cell transplantation

**DOI:** 10.1186/s40780-016-0052-9

**Published:** 2016-08-08

**Authors:** Yuki Tazawa, Akio Shigematsu, Kumiko Kasashi, Junichi Sugita, Tomoyuki Endo, Takeshi Kondo, Takanori Teshima, Ken Iseki, Mitsuru Sugawara, Yoh Takekuma

**Affiliations:** 1 Laboratory of Pharmacokinetics, Faculty of Pharmaceutical Sciences, Hokkaido University, Kita-12 Nishi-6, Kita-ku, Sapporo, Hokkaido 060-0812 Japan; 2 Department of Hematology, Hokkaido University Graduate School of Medicine, Sapporo, Japan; 3 Department of Pharmacy, Hokkaido University Hospital, Sapporo, Japan; 4 Education Research Center for Clinical Pharmacy, Faculty of Pharmaceutical Sciences, Hokkaido University, Sapporo, Japan

**Keywords:** Medium-dose etoposide, Allogeneic hematopoietic stem cell transplantation, Pharmacokinetics, Dose adjustment, Distribution volume

## Abstract

**Background:**

We investigated the pharmacokinetics of etoposide (ETP) to reduce the inter-individual variations of ETP concentrations in patients with acute leukemia who underwent allogeneic hematopoietic stem cell transplantation. We also carried out an in vivo study using rats to verify the dose adjustment.

**Methods:**

This study included 20 adult patients. ETP was administered intravenously at a dose of 15 mg/kg once daily for 2 days (total dose: 30 mg/kg) combined with standard conditioning of cyclophosphamide and total body irradiation. In an in vivo study using rats, ETP was administered intravenously at a dose of 15 mg/kg or an adjusted dose. The ETP plasma concentration was determined by using HPLC. The pharmacokinetic parameters were estimated by using a 1-compartment model.

**Results:**

The peak concentration (C_max_) of ETP and the area under the plasma concentration-time curve (AUC) of ETP differed greatly among patients (range of C_max_, 51.8 - 116.5 μg/mL; range of AUC, 870 - 2015 μg · h/mL). A significant relationship was found between C_max_ and AUC (*R* = 0.85, *P* < 0.05). Distribution volume (Vd) was suggested to be one of the factors of inter-individual variation in plasma concentration of ETP in patients (range of Vd, 0.13 - 0.27 L/kg), and correlated with Alb and body weight (*R* = 0.56, *P* < 0.05; *R* = 0.40, *P* < 0.05 respectively). We predicted Vd of rats by body weight of rats (with normal albumin levels and renal function), and the dose of ETP was adjusted using predicted Vd. In the dose adjustment group, the target plasma ETP concentration was achieved and the variation of plasma ETP concentration was decreased.

**Conclusion:**

The results suggested that inter-individual variation of plasma concentration of ETP could be reduced by predicting Vd. Prediction of Vd is effective for reducing individual variation of ETP concentration and might enable a good therapeutic effect to be achieved.

## Background

Allogeneic stem cell transplantation (allo-SCT) has been used to treat patients with hematological malignancies. High-dose intravenous etoposide (ETP) is commonly used with a standard conditioning regimen of cyclophosphamide (CY) and total body irradiation (TBI) [1–6]. However, it has been reported that the pharmacokinetic parameters of ETP were highly variable between individuals [7]. There have been many studies on the pharmacokinetics (PK) of ETP but only a few studies on leukemia patients who received high-dose ETP as a conditioning regimen and underwent allo-SCT [8, 9]. Moreover, the optimal dose of ETP has not been clarified.

In this study, we focused on PK and dose adjustment of ETP in adult patients with acute leukemia and also verified the dose adjustment in experimental rats based on the PK parameters to reduce large variations of plasma ETP concentration.

## Methods

### Patients and pharmacokinetic analysis

#### Patients

PK of ETP was evaluated in 20 patients who underwent allo-SCT using a conditioning regimen of medium-dose ETP + CY + TBI between April 2008 and January 2013 at Hokkaido University Hospital. A summary of the characteristics of the patients is shown in Table 1. Both the Protocol Review Committee and the Institutional Review Board of Hokkaido University Hospital approved the study. Written informed consent was obtained from all of the patients.

**Table 1:** Characteristics of the patients (*n* = 20)

		Mean ± S.D.	Median	Range
Age (year)		31.8 ± 7.0	31	18 – 44
Sex (male/female)	15/5			
Body Weight (kg)		67.0 ± 12.5	65.0	45.5 – 91.8
Body surface area (m^2^)		1.8 ± 0.2	1.8	1.4 – 2.1
Body mass index (kg/m^2^)		23.2 ± 3.3	23.1	15.5 – 28.8
Diagnosis				
ALL	17			
AML	2			
ANKL	1			
Disease status at SCT				
CR1	14			
CR2	1			
non CR	4			
relapse 1	1			
Donor				
MRD	6			
MUD	7			
MMRD	1			
MMUD	6			
Stem cell source				
Bone marrow	15			
Peripheral blood stem cells	3			
Umbilical cord blood	2			
GVHD Prophylaxis				
CSP + MTX	9			
TAC + MTX	11			
Laboratory data				
Alb (g/dL)		4.0 ± 0.4	4.0	2.8 – 4.9
T-pro (g/dL)		6.0 ± 0.4	5.8	5.4 – 7.0
BUN (mg/dL)		10.3 ± 3.0	10.5	5.0 – 15.0
Scr (mg/dL)		0.6 ± 0.2	0.6	0.3 – 1.0
T-bil (mg/dL)		0.7 ± 0.3	0.7	0.4 – 1.7
AST (IU/L)		35.0 ± 31.4	21.0	9.0 – 125
ALT (IU/L)		45.6 ± 54.6	32.0	5.0 – 251

*ALL* indicates acute lymphoblastic leukemia, *AML* acute myelogenous leukemia, *ANKL* aggressive NK cell leukemia, *SCT* stem cell transplantation, *CR* complete remission, *MRD* HLA–matched related donor, *MUD* HLA-matched unrelated donor, *MMRD* mismatched related donor, *MMUD* mismatched unrelated donor, *CSP* cyclosporin A, *MTX* methotrexate, *TAC* tacrolimus, *Alb* albumin, *T-pro* total protein, *BUN* blood urea nitrogen, *Scr* serum creatinine, *T-bil* total bilirubin, *AST* asparatate aminotransferase, *ALT* alanine aminotransferase

#### Conditioning regimen and graft-versus-host disease (GVHD) prophylaxis

All patients received the same conditioning regimen of medium-dose ETP + CY + TBI, which consisted of ETP at a dose of 15 mg/kg once daily administered intravenously (i.v.) over 3 h for 2 days (total dose: 30 mg/kg) and CY at 60 mg/kg once daily administered i.v. over 3 h for 2 days (total dose: 120 mg/kg) followed by 12 Gy of TBI delivered in 4 or 6 fractions for 2 or 3 days, as reported previously [10–13]. GVHD prophylaxis was provided with short-term methotrexate and cyclosporine (CSP) or tacrolimus (TAC) according to the physician’s selection.

#### Blood samples of patients

Blood samples were drawn before the start of ETP infusion (blank plasma) and at 1, 3, 6, 10, 24, 25, 27, 30, 34, 44, 68, and 92 h after the first infusion. The samples were collected into tubes containing heparin. The samples were centrifuged at 750 × g for 10 min at 4 °C to obtain plasma, and the plasma was frozen at -20 °C until analysis. All patients gave informed consent and agreed to the multiple blood sampling procedure.

#### Analytical procedure

ETP plasma concentration was determined by using HPLC. Analytical ETP was purchased from LKT Laboratories Inc. (St. Paul, MN, USA). It was dissolved in dimethyl sulfoxide (DMSO) (stock concentration: 20 mg/mL) and stored at -20 °C. Acetonitrile, dichloromethane, and methanol were of liquid chromatographic grade. Control plasma was provided by Japanese Red Cross Blood Center (Hokkaido, Japan) and stored at -20 °C. The internal standard, diphenyl hydantoin (DPH) was purchased from Wako Pure Chemical Industries, Ltd. (Osaka, Japan). ETP plasma concentration was determined by the method of kato et al. [14]. Briefly, 20 μL of DPH at a concentration of 100 μg/mL (in methanol), 1 mL of distilled water and 200 μL of plasma were added to a glass test tube with a screw cap. After 5 mL of dichloromethane had been added, the mixture was shaken for 15 min and then centrifuged at 750 × g for 5 min. Four mL of the dichloromethane layer was evaporated to dryness at 40 °C in a vacuum evaporator. The residue was redissolved in 200 μL of the mobile phase of HPLC and was subjected to HPLC. The injection volume of a sample was 40 μL. The HPLC system consisted of an L-7110 pump, L-7300 column oven, L-7420 UV-VIS detector, and D-2500 integrator (HITACHI, Tokyo, Japan). The column was an Inersil ODS-4 (100 mm × 2.1 mm i.d., 3 μm) (YOKOHAMARIKA CO., Yokohama, Japan). A mobile phase containing methanol/distilled water/acetonitrile (42.7: 55: 2.3, v/v/v) was used at a flow rate of 0.4 mL/min. The detector was monitored at 229 nm.

#### Pharmacokinetic analysis

The pharmacokinetic parameters were estimated by using a 1-compartment model. The peak concentration (C_max_) and the trough concentration (C_min_) of ETP in plasma were obtained directly from the analytical data. The volume of distribution (Vd) was calculated as Dose/C_0_ (C_max_). The elimination rate constant (K_el_) was calculated by log-linear regression of ETP concentration data during the elimination phase. The clearance (CL) was calculated as K_el_ × Vd. The area under the plasma concentration-time curve (AUC) was calculated by the trapezoidal rule. Mean values of Vd on the first day and second day were used for subsequent investigation.

### Experimental animals and pharmacokinetic analysis

#### Animals and treatment

Male Wistar rats were obtained from Hokudo Co., Ltd. (Sapporo, Japan). The experimental protocols were reviewed by the Animal Care Committee in accordance with the Guide for the Care and Use of Laboratory Animals. ETP for intravenous infusion was purchased from Sandoz (Tokyo, Japan). ETP was diluted in normal saline. ETP solution was administrated intravenously at a dose of 15 mg/kg. At each experimental time point (before the start of ETP infusion (blank plasma) and at 0.017, 0.05, 0.25, 0.75, 1.5, 3, and 6 h after infusion), rats were anesthetized with diethyl ether, and whole blood was collected from the jugular vein. Plasma was obtained by centrifugation at 750 × g for 10 min at 4 °C. The rats were killed by exsanguination after blood collection. ETP plasma concentration was determined by HPLC as described above. PK parameters were determined as described above.

#### Statistical analysis

Student’s *t*-test was used to determine the significance of differences between two group means. Pearson’s test was used to determine correlations. Predictability of Vd was calculated with stepwise regression analysis using JMP® 12 Pro (SAS Institute Inc., Cary, NC, USA). Statistical significance was defined as *P* < 0.05.

## Results

### Results for the patients

#### Pharmacokinetic analysis of ETP in patients

**Fig. 1:**
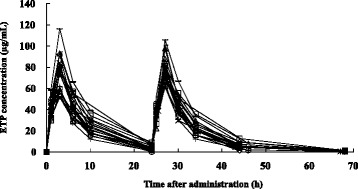
Plasma concentration of ETP in patients after i.v. administration of ETP over 3 h at a dose of 15 mg/kg once daily for 2 days (*n* = 20)

The plasma concentration versus time curve and the pharmacokinetic parameters after intravenous administration of ETP are shown in Fig. 1 and Table 2, respectively. Mean C_max_ on the first day was 74.9 μg/mL (median: 77.4, range: 51.8 - 116.5 μg/mL). Mean AUC_0-92h_ was 1332 μg · hr/mL (median: 1282, range: 870 - 2015 μg · h/mL). Mean values of Vd on the first day and second day were 0.20 L/kg (median: 0.20, range: 0.13 - 0.28). A significant relationship was found between C_max_　(day1) and AUC_0-92h_ (*R* = 0.85, *P* < 0.05). Vd was correlated with Alb and body weight (*R* = 0.56, *P* < 0.05; *R* = 0.40, *P* < 0.05 respectively).

**Table 2:** Pharmacokinetic parameters of ETP after administration at a dose of 15 mg/kg once daily over 3 h for 2 days in patients (*n* = 20)

Parameter	Day 1			Day 2		
	Mean ± S.D.	Median	Range	Mean ± S.D.	Median	Range
Cmax (μg/mL)	74.9 ± 17.0	77.4	51.8 – 116.5	77.5 ± 12.9	75.8	61.6 – 106.0
Cmin (μg/mL)	3.9 ± 2.5	3.7	0.2 – 9.1	6.2 ± 3.7	5.4	1.6 – 16.1
Vd (L/kg)	0.20 ± 0.05	0.19	0.12 – 0.29	0.20 ± 0.04	0.20	0.14 – 0.27
Kel (h^–1^)	0.15 ± 0.04	0.14	0.10 – 0.29	0.14 ± 0.03	0.14	0.07 – 0.20
CL (mL/h/kg)	30.9 ± 14.5	24.5	14.2 – 72.6	29.0 ± 8.5	27.4	11.3 – 45.5
AUC0-24 h or AUC24–44 h (μg・h/mL)	634 ± 154	611	423 – 1021	612 ± 126	586	424 – 852
AUC0-92 h (μg・h/mL)	1332 ± 315	1282	870 – 2015			

*Vd* indicates volume of distribution, *Kel* elimination rate constant, *CL* clearance, *AUC* area under the plasma concentration–time curve

### Results of experiments using rats

#### Pharmacokinetic analysis of ETP in rats

We investigated the pharmacokinetic parameters in rats (with normal Alb levels and renal function). The experimental rats were divided into 3 groups based on the age of rats [5 weeks (control), 7 weeks and 10 weeks] and were intravenously administered ETP at a dose of 15 mg/kg. Table 3 shows the pharmacokinetic parameters of rats after infusion. C_max_ and AUC were significantly higher in the groups of 7 weeks and 10 weeks than in the group of 5 weeks (control). Vd in the group of 10 weeks were lower than those in the group of 5 weeks. Kel was not significantly different among the 3 groups of rats.

**Table 3:** Pharmacokinetic parameters of ETP after intravenous administration at a dose of 15 mg/kg in rats

	5 weeks (control)	7 weeks	10 weeks
	120 – 150 g	220 – 230 g	320 – 355 g
AUC (μg・h/mL)	12.8 ± 0.33	15.8 ± 1.21*	20.4 ± 3.01*
Cmax (μg/mL)	49.0 ± 9.73	51.7 ± 4.41	71.8 ± 7.57*
Vd (L/kg)	0.32 ± 0.07	0.29 ± 0.03	0.21 ± 0.02*
Kel (h^–1^)	1.72 ± 0.26	1.80 ± 0.10	1.84 ± 0.38
CL (L/h/kg)	0.54 ± 0.12	0.52 ± 0.06	0.39 ± 0.10*

Each value is the mean ± S.D. of 3 - 4 measurements

*Significantly different from control at *p* <0.05

#### Dose adjustment of ETP in rats

**Fig. 2:**
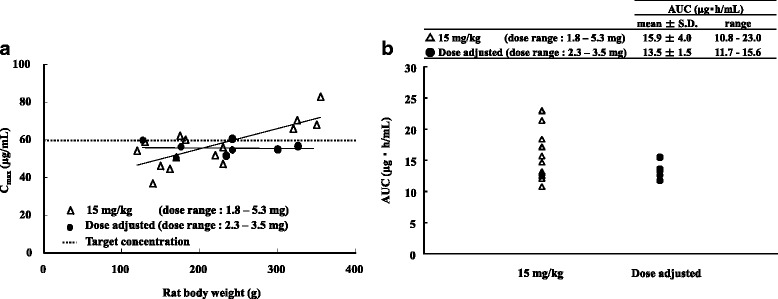
Comparison of (**a**) Cmax of ETP and (**b**) AUC of ETP after intravenous administration of ETP at a dose of 15 mg/kg (△) and at the adjusted dose (●) in rats

There was a positive correlation between body weight and Vd of ETP in rats (linear regression equation: Vd (L) = 0.0001 × body weight (g) + 0.0259, *R* = 0.82, *P* < 0.05). Therefore, we predicted Vd from the body weights of rats and calculated the dose by the following formula: dose (mg) = Vd (L) × C_max_ (μg/mL) to achieve target C_max_ (60 μg/mL). We set a target ETP concentration to 60 μg/mL because the mean C_max_ of ETP in the group of rats administered 15 mg/kg was 57 μg/mL. C_max_ of ETP in the group of rats administered 15 mg/kg increased with increase in body weight (Fig. 2 (a)). On the other hand, the group of rats administered the adjusted dose achieved the target C_max_ (Fig. 2 (a)). Moreover, when comparing the AUC at this time, the variation of ETP concentration was decreased in the adjustment group (Fig. 2 (b)).

## Discussion

Although the standard conditioning regimen of CY + TBI has been widely used before allo-SCT, the rate of mortality due to relapse is high and the results of treatment are not satisfactory [15–20]. Therefore, various intensified conditioning regimens, some of which used ETP combined with CY + TBI, have been developed. Many studies including studies in which ETP (60 mg/kg) was combined with CY and TBI (ETP + CY + TBI) showed a low relapse rate but high rates of toxicity and transplant-related mortality [1–6]. We previously reported excellent outcomes for patients who received a medium-dose ETP (30 mg/kg) + CY + TBI regimen at Hokkaido University Hospital in Japan [10, 11] and superior survival to that in patients who received CY + TBI retrospectively [12]. We also conducted a prospective phase II study. In that study, 1-year overall survival was 80.8 % (95 % *Cl* = 66.0 - 88.7 %). No patient died within 100 days post-SCT. The cumulative incidences of relapse and non-relapse mortality at 1-year post SCT were 10.0 and 14.0 %, respectively [13]. These data indicated that the addition of ETP was important for outcomes; however, there have been no study on PK of medium-dose ETP in adult patients with leukemia.

In this study, we focused on the PK of ETP with the aim of establishing the optimal dosage of ETP. Firstly, the plasma concentration and pharmacokinetic parameters of ETP in patients who received medium-dose ETP were determined. In most studies, 2-compartment models were used for PK analysis of ETP [21, 22]. However, we consider that α-phase of etoposide is almost completed at the end of the administration because etoposide administered over 3 h and a semi-logarithmic plot of plasma concentration versus time appear as a single straight line. Therefore, we used 1- compartment model for analysis.

It was found that the plasma concentrations of ETP differed greatly among patients (Fig. 1, Table 2). The plasma concentrations of ETP should normally be about the same in all patients. Therefore, factors that account for the inter-individual variation in the plasma concentration of ETP were investigated in this study. It has been reported that the steady-state concentration and AUC of continuous infusion of ETP were related to its toxicity [7, 23]. In this study, a significant relationship was found between C_max_ and AUC_0-92h_ (*R* = 0.85, *P* < 0.05). Therefore, we focused on factors that cause the inter-individual variation of C_max_. Individual differences in C_max_ are considered to be due to variation of Vd because Vd is calculated by the following equation: *Vd* = Dose/C_max_. We found that Vd was correlated with Alb and body weight. Protein binding is important for PK of ETP. ETP is highly bound to Alb in plasma and the ratio of protein binding is 93 % [24]. Stewart et al. reported that unbound ETP was significantly increased in cancer patients compared with that in normal volunteers [25]. These alterations in protein binding were significantly related to Alb [25]. A relationship between the ETP binding ratio and Alb was reported by Schwinghammer et al. (*R* = 0.57, *P* = 0.02) [26].

About 35 % of the administered dose of ETP is excreted into urine as the parent drug [27]. ETP clearance was significantly correlated with serum creatinine (Scr) in previous studies [28, 29]. In the present study, the renal function of 20 patients is normal range (Scr 0.3 - 1.0 mg/dL). Therefore, we considered that Vd is important for patients with normal renal function. The study by Krogh-Madsen [22], baseline white blood cell count (bWBC) and sex influenced the PK of ETP. However, in this study, no correlation of bWBC and sex on PK of ETP. These results show that the variability of AUC could be reduced to adjust dosages by predicted Vd in patients with normal renal function.

We have investigated the study using rats whether to reduce the variation of ETP concentration by dose adjustment by prediction of Vd. Our in vivo study in rats suggested that increase of ETP plasma concentration was mainly associated with increase of body weight (Table 3). In addition, body weight of rats was strongly correlated with Vd (*R* = 0.82, *P* < 0.05). Therefore, we predicted Vd by only body weight of rats and calculated the dose of ETP so as to achieve a target ETP plasma concentration (60 μg/mL). As a result, the group of rats with dose adjustment achieved the target ETP plasma concentration and the variation of plasma ETP concentration was decreased. These results indicate that body weight is very important for pharmacokinetic parameters of ETP, especially Vd.

In the investigation using rats, it was shown that body weight is very important for Vd and that Vd can be predicted by body weight. In general, the body surface area (BSA) is used in dose adjustment of chemotherapy. However, there was high inter-individual variation in plasma concentration of ETP, even if dose of ETP was adjusted based on BSA [21]. In addition, body weight-based dose has been widely used in conditioning regimens [8, 9, 19]. Therefore, we use body weight to determine the dosage.

In clinical investigations, we focused on only Vd because there was a strong correlation between C_max_ and AUC. However, K_el_ is critical for estimating ETP plasma concentration as well as Vd. In addition, the target ETP plasma concentration in medium dose ETP therapy has not been clarified. According to our preliminary analysis, ETP plasma concentration ≥ 75.6 μg/mL was associated with a high mortality rate. However, correlations between results of pharmacokinetic analysis and clinical outcomes were not sufficient in this study due to the small sample size. Further studies are needed to establish the optimal dose of ETP and confirm correlations between pharmacokinetic parameters of ETP and clinical outcomes in different patient populations.

## Conclusions

The results suggested that inter-individual variation of plasma concentration of ETP could be reduced by predicting Vd. Prediction of Vd is effective for reducing individual variation of ETP concentration and might enable a good therapeutic effect to be achieved.

## Abbreviations

Alb, albumin; ALL, acute lymphoblastic leukemia; allo-SCT, allogeneic stem cell transplantation; ALT, alanine aminotransferase; AML, acute myelogenous leukemia; ANKL, aggressive NK cell leukemia; AST, asparatate aminotransferase; AUC, area under the plasma concentration-time curve; BUN, blood urea nitrogen; CI, confidence intervals; CL, Clearance; C_max_, peak concentration; C_min_, trough concentration; CR, complete remission; CSP, cyclosporine; CY, cyclophosphamide; DMSO, dimethyl sulfoxide; DPH, diphenyl hydantoin; ETP, etoposide; GVHD, graft-versus-host disease; HPLC, high-performance liquid chromatography; i.v., intravenously; K_el_, elimination rate constant; MMRD, mismatched related donor; MMUD, mismatched unrelated donor; MRD, HLA-matched related donor; MTX, methotrexate; MUD, HLA-matched unrelated donor; PK, pharmacokinetics; RMSE, root mean squared error; Scr, serum creatinine; TAC, Tacrolimus; TBI, total body irradiation; T-bil, total bilirubin; T-pro, total protein; Vd, distribution volume
